# Reducing the Social Gradient in Uptake of the NHS Colorectal Cancer Screening Programme Using a Narrative-Based Information Leaflet: A Cluster-Randomised Trial

**DOI:** 10.1155/2016/3670150

**Published:** 2016-03-16

**Authors:** Lesley M. McGregor, Christian von Wagner, Wendy Atkin, Ines Kralj-Hans, Stephen P. Halloran, Graham Handley, Richard F. Logan, Sandra Rainbow, Steve Smith, Julia Snowball, Mary C. Thomas, Samuel G. Smith, Gemma Vart, Rosemary Howe, Nicholas Counsell, Allan Hackshaw, Stephen Morris, Stephen W. Duffy, Rosalind Raine, Jane Wardle

**Affiliations:** ^1^Department of Epidemiology and Public Health, University College London, London WC1E 7HB, UK; ^2^Department of Surgery & Cancer, Imperial College London, London SW7 2AZ, UK; ^3^Department of Biostatistics, King's Clinical Trials Unit, Institute of Psychiatry, Psychology and Neuroscience, King's College London, London SE5 8AF, UK; ^4^NHS Bowel Cancer Screening Southern Programme Hub, Surrey Research Park, Guildford, Surrey GU2 7YS, UK; ^5^Faculty of Health and Medical Sciences, University of Surrey, Guildford, Surrey GU2 7TE, UK; ^6^NHS Bowel Cancer Screening North East Programme Hub, Gateshead Health NHS Foundation Trust, Queen Elizabeth Hospital, Gateshead NE9 6SX, UK; ^7^NHS Bowel Cancer Screening Eastern Programme Hub, Nottingham University Hospitals, Nottingham NG7 2UH, UK; ^8^NHS Bowel Cancer Screening London Programme Hub, Northwick Park and St Mark's Hospitals, Harrow, Middlesex HA1 3UJ, UK; ^9^NHS Bowel Cancer Screening Midlands and North West Programme Hub, Hospital of St Cross, Barby Road, Rugby CV22 5PX, UK; ^10^Department of Applied Health Research, University College London, London WC1E 7HB, UK; ^11^Wolfson Institute of Preventive Medicine, Queen Mary University of London, London EC1M 6BQ, UK; ^12^Cancer Research UK & UCL Cancer Trials Centre, Cancer Institute, University College London, London W1T 4TJ, UK

## Abstract

*Objective*. To test the effectiveness of adding a narrative leaflet to the current information material delivered by the NHS English colorectal cancer (CRC) screening programme on reducing socioeconomic inequalities in uptake.* Participants*. 150,417 adults (59–74 years) routinely invited to complete the guaiac Faecal Occult Blood test (gFOBt) in March 2013.* Design*. A cluster randomised controlled trial (ISRCTN74121020) to compare uptake between two arms. The control arm received the standard NHS CRC screening information material (SI) and the intervention arm received the standard information plus a supplementary narrative leaflet, which had previously been shown to increase screening intentions (SI + N). Between group comparisons were made for uptake overall and across socioeconomic status (SES).* Results*. Uptake was 57.7% and did not differ significantly between the two trial arms (SI: 58.5%; SI + N: 56.7%; odds ratio = 0.93; 95% confidence interval: 0.81–1.06; *p* = 0.27). There was no interaction between group and SES quintile (*p* = 0.44).* Conclusions*. Adding a narrative leaflet to existing information materials does not reduce the SES gradient in uptake. Despite the benefits of using a pragmatic trial design, the need to add to, rather than replace, existing information may have limited the true value of an evidence-based intervention on behaviour.

## 1. Introduction

The English Bowel (colorectal cancer, CRC) Screening Programme involves sending a guaiac Faecal Occult Blood test (gFOBt) to the homes of men and women aged 60–74 every two years. It is now widely accepted that gFOBt screening reduces cancer-specific mortality [1–3], but uptake of only 54% greatly undermines the potential public health benefits of the programme [4]. Importantly, there are also inequalities in screening uptake by socioeconomic status (SES) which are likely to exacerbate existing inequalities in CRC outcomes.

Recently, there has been interest in the extent to which socioeconomic inequalities are modified or maintained by the information sent with invitation materials. Health communication is often didactic (factsheets, charts, etc.), targeting a more “rational” thinking process. However, this presentation may contradict preexisting beliefs and behaviours and subsequently elicit defensive processing, such as counterarguing or disengagement, allowing the individual to preserve their positive self-image, while undermining the effectiveness of the health message [5, 6].

Narrative information is thought to target a more “heuristic/affective” thinking process and, therefore, may be considered a less direct confrontation of a person's health beliefs, thereby reducing initial defensive processing [7, 8]. By additionally promoting mental imagery and providing role models of desired behaviours, narratives are thought to be more engaging [7, 9].

In a recent qualitative study exploring people's responses to narrative information about colorectal cancer screening information, respondents described narrative information as helpful, making the information more vivid, relevant, and subsequently reassuring [10]. Several US-based studies have found narrative information to be associated with stronger intention to take part in cancer screening than nonnarrative information (e.g., [11, 12]), and there is some evidence of higher uptake of cancer screening tests, particularly among more socioeconomically deprived and ethnically diverse groups [13, 14]. However, there is currently no research within the UK CRC screening programme exploring the potential of narrative information on reducing the socioeconomic gradient in uptake of the English CRC screening programme.

We developed a narrative leaflet, containing quotes and stories of the CRC screening experience from previous participants, as part of a wider project to test four separate, theoretically driven interventions to reduce socioeconomic inequalities in CRC screening uptake [15]. The other interventions included a “gist” based leaflet, which simplified the information contained in the standard information materials in a format suitable for those with low literacy skills; both the gist and narrative leaflets were designed as a supplement to the standard information provided to patients. In addition, amendments to the standard information materials were made for two further interventions: a GP endorsed invitation letter [16] and an enhanced reminder letter. The impact of each of the four interventions on CRC screening uptake overall and across socioeconomic status (SES) groups is published elsewhere [15]. In this paper we describe in more detail the results of the narrative leaflet specifically, considering its influence on uptake by various population characteristics (e.g., age, gender) and if this changes across SES.

In the initial evaluation of the leaflet, we conducted a questionnaire study with over 1200 individuals approaching the age of screening and observed that adding the narrative leaflet to standard screening information enhanced intention to screen, particularly by strengthening anticipated peace of mind from screening, enhancing feelings of vulnerability to colorectal cancer without screening, reducing perceived disgust with the screening procedure, and strengthening the belief that screening can reduce the chance of dying from the disease [17]. Here, we report the results of a national randomised controlled trial to test the effectiveness of this supplementary narrative leaflet on reducing socioeconomic inequalities in uptake of the CRC screening programme.

## 2. Methods

A two-arm cluster randomised controlled trial (ISRCTN74121020) based on the day of the week that materials were mailed compared uptake between those who were sent the standard NHS CRC screening information material (SI) and those who were sent the standard material plus the narrative-based information leaflet (SI + N). Ethical approval for this study was awarded by the National Research Ethics Service Committee London-Harrow in September 2012 (REC ref: 12/LO/1396) and an Independent Data Monitoring & Ethics Committee (IDMEC) monitored the progress of the trial.

### 2.1. Settings and Participants

**Figure 1:**
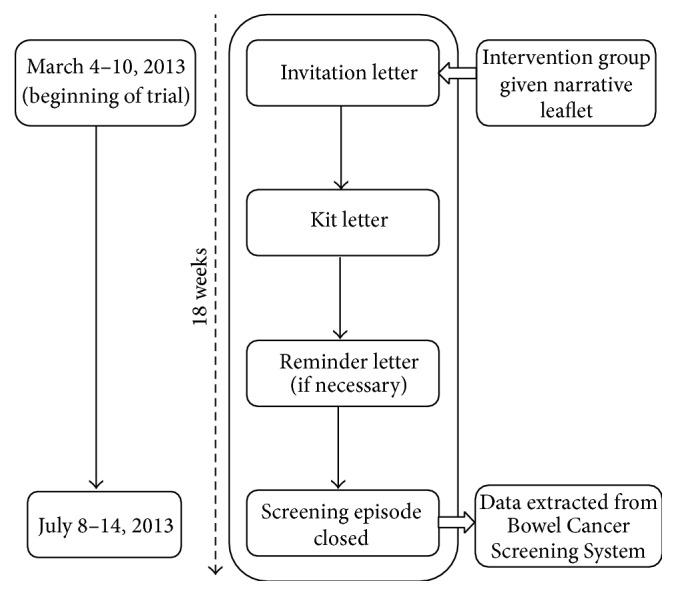
Organisation and schedule of the national trial.

In England, the CRC screening programme invites men and women who are registered with a GP to complete a gFOBt every two years from the age of 60 (up to 74 years). The programme is coordinated through 5 hubs (Midlands and North West, Southern, London, North East, and Eastern), where each mails screening invitations to approximately 60,000 eligible people per month (prevalent and incident screening rounds combined; “prevalent” relates to individuals who are being invited for the first time or have not accepted previous invitations to take part in the CRC screening programme and “incident” relates to individuals who have participated in screening at least once before). The invitation letter is mailed with an information booklet (“Bowel Cancer Screening: the Facts”; online version is available at http://www.cancerscreening.nhs.uk/bowel/publications/information-leaflets.html) (standard information SI). The gFOBt test kit and instruction leaflet is mailed 8–10 days later (see Figure 1).

Completing the gFOBt involves taking two small samples from three separate bowel motions over a 10-day period. The CRC screening programme in England does not impose any dietary restrictions for completion of the test [18]. The samples are collected using provided cardboard sticks and applied to a specific section of guaiac paper on the test kit. The test kit is then mailed in a hygienic, prepaid envelope back to the hub for processing. Within 2 weeks, result letters are sent by the hub to the individual and their General Practitioner (GP). For those with a “normal” result, no further action is taken and they are invited to participate again 2 years later. If the returned test kit was “spoiled,” had a technical fail, or produced an unclear result, a second test kit is sent for completion. Those with an abnormal test result are sent an invitation to attend a follow-up test (i.e., colonoscopy) at their local screening centre.

A reminder letter is sent if a completed kit is not returned within 4 weeks. If there is no return within a further 13 weeks, the “screening episode” is closed for that round of screening. The next round of the screening invitation process starts again 2 years later (up to the age of 74).

This study was integrated into the usual running of each hub over a 10-day period (March 4–15, 2013). For two hubs, the printing and distribution of invitation material is conducted in-house through a system operated by Health and Social Care Information Centre (HSCIC). For the remaining three hubs, this activity is contracted out to REAL Digital International, Croydon, UK (RDI). Both CfH and RDI agreed to accommodate the inclusion of an additional leaflet as per study design.

### 2.2. Intervention

#### 2.2.1. Control Group: Standard Information (SI) Booklet

As per the CRC screening programme protocol, each person was mailed an NHS envelope containing a one-page invitation letter and “The Facts” booklet. Approximately one week later they were mailed a gFOBt kit with a standard instructions leaflet. If no response was received within 4 weeks, a standard reminder letter was sent.

#### 2.2.2. Intervention Group: Standard Information Booklet + Narrative Leaflet (SI + N)

The initial NHS envelope contained, in addition to the invitation letter and “The Facts” booklet, an additional narrative leaflet (see Supplementary Appendix 1 in Supplementary Material available online at http://dx.doi.org/10.1155/2016/3670150). Twenty individuals who had previously taken part in the NHS BCSP (17 had received “abnormal” gFOBt results, with 8 diagnosed with CRC) were interviewed about their CRC screening experience. Key content for the leaflet was sourced from the interview transcripts. Photographs of the interviewees were presented alongside selected quotes (e.g., “*My first thought about the test kit was that it was going to be messy, but it didn't actually turn out to be.*”) and individual summarised “stories.” Two focus groups with members of a local community centre provided feedback on an early version of the leaflet. A subsequent amended version was then sent to the interviewees for review: no further changes were made. An evaluation of the leaflet using the Comprehensibility Assessment of Materials (SAM + CAM) questionnaire confirmed that the design, content, and layout of the final draft were of a “superior standard” [19]. In addition, a Flesch Reading Ease score of 66.2 was calculated for the text within the leaflet, suggesting it was of a “standard” reading level, easily understood by 13–15 year olds (American grade 9) [20, 21]. The final leaflet was presented in an A4 trifold format and was entitled “Bowel Cancer Screening: People's Stories.” For further details on leaflet development and evaluation see McGregor et al. 2015 [17].

### 2.3. Randomisation

The randomisation schedule was provided to CfH, RDI, and each corresponding hub in advance of the study commencing. Each schedule was derived from a random number generator, whereby 10 numbers corresponding to the 10-day study period were generated. Days assigned numbers 1–5 and 6–10 were designated either control (SI) or intervention days (SI + N).

The hubs, CfH, and RDI could not be blind to group allocation, but a lack of direct contact with participants meant that any associated bias was minimised. Participants were unaware of their involvement in a randomised study and therefore blind to group allocation (unless they communicated with an eligible person assigned to the other group during this study).

### 2.4. Outcome Measures

*Primary Outcome*. The primary outcome was the number of people “adequately screened” within each group (SI versus SI + N). To be classified as “adequately screened,” an individual must have completed and returned a gFOBt kit within 18 weeks of being sent the invitation letter (see Figure 1), with a definitive “normal” (no further investigation required) or “abnormal” (requiring referral for further investigation, usually colonoscopy) screening test result at the time of data extraction.

The Index of Multiple Deprivation (IMD) scores of each address was the basis for estimating deprivation. IMD assesses deprivation at the small area level (i.e., lower layer superoutput areas (LSOA) covering about 1500 people) and takes into account seven deprivation domains: income, employment, health and disability, education, skills and training barriers to housing and services, crime, and living environment. Each domain is scored separately and they are combined to produce an IMD score for each LSOA. Postcodes are linked to LSOAs and therefore to IMD scores. IMD scores have been shown to be correlated with individual markers of SES [22]. Within this study, national data for IMD quintiles were applied to the obtained scores, with postcodes falling in the first quintile denoting the most socially advantaged group (higher SES) and those in the fifth quintile the most socially disadvantaged group (lower SES).

The effect of screening round was investigated with individuals classified as either prevalent first time invitees, prevalent previous nonresponders, or incident episodes (participated at least once previously). Age, gender, and hub were additionally considered potential moderators. An additional outcome was the time taken to return a completed gFOBt kit.

### 2.5. Statistical Considerations

The target sample size was based on achieving a reduction in the SES gradient associated with screening uptake. We assumed a fixed proportional effect in each hub and estimated an average increase of 3 percentage points, based on increasing uptake by 5 percentage points in the lowest (fifth) IMD quintile (most socially disadvantaged group) and 1 percentage point in the highest (first) quintile, giving an overall 1-2-3-4-5 percentage point difference by quintile using the method of Brentnall and colleagues [23]. This estimate was drawn from the outcomes that were considered feasible in research aiming to increase screening uptake [24].

The final calculation assumed the composition of the hub that required the largest sample (North West) to ensure a sufficiently large number of people. Because the study randomised by day and there is variation in the number of invitations sent per day, an inflation factor of 1.7 was included. With 90% power and 5% statistical significance, 46,000 individuals (23,000 per arm) were required to detect a 1-2-3-4-5 percentage point difference in uptake in the least to most deprived IMD quintile, respectively. Due to the volume of invitations sent out by each hub during a working week (70,000–80,000), this sample would have been achieved within only 5 days, but such small number of clusters would have a high risk of bias. The intervention therefore ran for 10 days, providing a sample of approximately 140,000–160,000, to achieve enough clusters to avoid bias [25].

The primary outcome was analysed by logistic regression in a univariable model and then a multivariable model adjusting for age, gender, hub and screening round. *p* values and 95% confidence intervals calculated using conservative variance estimation to allow for the potential clustering effects in randomisation [26]. The association between the proportion of people adequately screened and SES was assessed by including an interaction term between trial arm and IMD quintile in models. The association was also investigated by stratifying according to age, gender, screening round, and hub. Analysis was performed on an intention-to-treat basis, using SAS v9.3 (SAS Institute Inc., Cary, NC, USA) and Stata v12.1 (StataCorp LP, College Station, TX, USA).

HSCIC organised for the relevant pseudoanonymised data to be extracted from the Bowel Cancer Screening System and transferred to one coordinating hub (Southern). At this hub, the data were cleaned and further anonymised before being securely transferred to the research team for analysis.

### 2.6. Assessment of Concurrent Initiatives

An audit of current and imminent bowel cancer screening research and health promotion activities within England was carried out and regularly updated throughout the course of this research study. This audit served to highlight any possible confounding factors to consider in our analysis and involved regular contact with key informants including Quality Assurance Reference Centre (QARC) directors, Specialist Screening Practitioners (SSPs), BCSP managers, and representatives from the National Awareness and Early Diagnosis Initiative (NAEDI), Cancer Research Network, and Strategic Clinical Network.

## 3. Results

**Figure 2:**
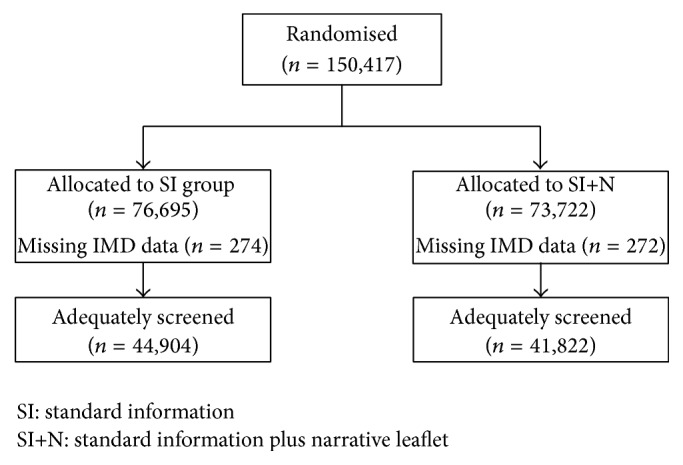
Flowchart of participation.

Between March 4 and 15, 2013, 150,417 individuals were allocated to either the control group (SI: 76,695) or the intervention group (SI + N: 73,722) (see Figure 2). Baseline characteristics were generally well-balanced, although there was a higher proportion of incident screens in the control group, and randomisation resulted in two hubs (Southern and Eastern) having a disproportionate number of people in each trial arm (Table 1).

The median time (range) to return a kit was 26 days (10–126) and 26 days (11–126) for the SI and SI + N groups, respectively. Spoilt kits were returned by only 1,204 individuals (SI: 595; SI + N: 609). Overall, more than half (57.7%; 86,726) of those invited returned a gFOBt kit within 18 weeks of their invitation that led to a “definitive” screening test result of either “normal” or “abnormal” within the timeframe of the study (i.e., were “adequately screened”).

The intervention arm (SI + N = 56.7%; 41,822) had a proportion of adequately screened individuals 1.8 percentage points lower than the control arm (SI = 58.5%; 44,904); this reduction was not statistically significant (unadjusted odds ratio (OR) = 0.93, 95% confidence interval (CI) 0.81–1.06, *p* = 0.27; adjusted OR = 1.00, 95% CI 0.96–1.03, *p* = 0.80).

For each trial arm, the proportion of adequately screened individuals within each IMD quintile decreased as the level of deprivation increased (SI: 66.8% to 46.0%; SI + N: 64.6% to 42.4%; Table 2). There was no evidence of a reduction in the social gradient in the intervention arm (interaction between the trial intervention and deprivation quintile: unadjusted *p* = 0.44, adjusted *p* = 0.11); however, a lower proportion of individuals were adequately screened in the intervention arm than the control arm within the most deprived quintile (unadjusted OR = 0.86, 95% CI 0.74–1.00, *p* = 0.05; adjusted OR = 0.92, 95% CI 0.86–0.98, *p* = 0.02).

There was no difference in the overall proportion of individuals adequately screened between the trial groups by age at invitation (median age = 65 yrs; Table 3). The proportion screened was generally lower in younger individuals and decreased with deprivation in both arms. There was no evidence of an association between the trial arm and deprivation quintile on the proportion screened in either age group (unadjusted interaction: <65 yrs, *p* = 0.37; ≥65 yrs, *p* = 0.67; adjusted interaction: <65 yrs, *p* = 0.07; ≥65 yrs, *p* = 0.88).

There was no difference in the overall proportion adequately screened between the trial arms by gender (Table 4). The proportion screened was generally lower in men and decreased with deprivation in both arms, but with no arm-by-deprivation quintile interaction for men (unadjusted *p* = 0.33; adjusted *p* = 0.05) or women (unadjusted *p* = 0.68; adjusted *p* = 0.60).

The proportion adequately screened was lower in people who had not previously taken part in CRC screening and decreased with deprivation in both arms (Table 5), but there was no evidence of an association between trial arm and deprivation quintile in either screening round (unadjusted interaction: prevalent first-time invitees, *p* = 0.30; prevalent previous nonresponders, *p* = 0.59; incident episodes, *p* = 0.70) (adjusted interaction: prevalent first-time invitees, *p* = 0.30; prevalent previous nonresponders, *p* = 0.71; incident episodes, *p* = 0.60).

There was no difference in the overall proportion of individuals adequately screened between the trial groups in each hub (Table 6). There was no interaction with deprivation score in any of the hubs (unadjusted interaction: Midlands and North West, *p* = 0.93; Southern, *p* = 0.68; London, *p* = 0.84; North East, *p* = 0.24; Eastern, *p* = 0.69) (adjusted interaction: Midlands and North West, *p* = 0.61; Southern, *p* = 0.60; London, *p* = 0.09; North East, *p* = 0.13; Eastern, *p* = 0.51).

As part of the concurrent initiative survey, we received details of 62 local health promotion activities and 17 research projects being undertaken at the same time as this trial. None of these initiatives were limited to occurring on the same days the narrative leaflet was sent out.

## 4. Discussion

This study assessed the impact of the addition of a narrative-based leaflet to existing information on inequalities in uptake of CRC screening in England. The leaflet was designed to reduce barriers to uptake but it neither increased the overall number of people returning a completed gFOBt kit nor decreased the associated SES gradient. In fact, this study appeared to produce negative results at various points. However, we found that this was due to the imbalance in screening round, with a higher proportion of incident screens in the control group; this is because ~85% of incident screens were adequately screened, compared to only ~50% of prevalent first-time invitees and ~14% of prevalent previous nonresponders. After adjusting for baseline characteristics, we found no evidence of an overall effect in either direction (OR = 1.00, 95% CI 0.96–1.03, *p* = 0.80) nor a general trend within subgroups. The narrative leaflet, therefore, had neither a positive nor negative impact on uptake. Overall, the use of this supplementary intervention was unsuccessful and it is unlikely that the lack of effect can be ascribed to concurrent initiatives.

Our study benefited from a pragmatic design, including access to a very large sample size, with no exclusion criteria, and the real-life applicability of the intervention within an established CRC screening programme [27]. However, our results run counter to previous research in the USA which demonstrated the potential effectiveness of narrative information to increase CRC screening uptake [13, 14]. It is possible that, within a UK setting, narratives are considered to be credible and useful for CRC screening decision making [10] but are, in reality, not an effective intervention to increase uptake. However, upon reflection, our negative results may also have stemmed from the need to fit the intervention into the existing screening programme structure. This requirement may have limited the impact of the narrative in two ways.

Firstly, it meant that the additional information leaflet could only be inserted at the preinvitation stage; due to logistics of the packing equipment used in two hubs, inclusion of the leaflet with the second mail out (test kit and instructions) was not possible. Although research indicates the importance of a preinvitation contact point at improving uptake [28], adding a narrative leaflet addressing barriers to screening at this stage might not have been the most appropriate time-point. Although we have previously shown the leaflet to increase intention to complete screening in screening naïve individuals [17], one may argue that individuals receiving repeat invitations may not engage with the information sent at the preinvitation stage to the same extent as those invited for the first time. Within this study the majority of invitees were incident screens and, therefore, simply may not have engaged with the leaflet. Had the leaflet accompanied the test kit, individual may have been more likely to attend to it and it may have additionally acted as a point-of-choice prompt and subsequently had a stronger impact on actual test completion [29]. The most effective positioning of the narrative leaflet requires further investigation.

Secondly, the narrative leaflet had to be added to, rather than replace, existing informational literature. Despite the brevity of the narrative leaflet, by adding it to existing information material, the total amount of written material received by the individual increased. This might have undermined the goal of making the screening offer more visible to lower-literacy individuals. The impact of a “stand-alone” narrative leaflet at different points in the invitation pathway and in comparison to existing materials therefore needs to be examined.

In addition, the provision of narrative information in different formats (e.g., web, video) which integrate oral and nonverbal communication methods, thus allowing more narrative information to be communicated, and perhaps in a more engaging manner, should also be tested. Previous research has also suggested a benefit of using either a targeted or tailored approach in health communication materials (e.g., [30, 31]). Had multiple variations of the leaflet been developed to specifically target, for example, those who were being invited for the first time and previous nonresponders separately, the results may have been more positive. However, within a national programme, the application of targeted and tailored materials is complex. Future research is needed to investigate ways to implement such interventions on a national scale.

Further limitations of the study include the marker of SES used to depict the gradient. While the Index of Multiple Deprivation (IMD) is a comprehensive measure of area deprivation, it does not include personal circumstances and therefore individuals may not be as affluent or as disadvantaged as their postcode suggests.

The current study does not support the inclusion of a narrative leaflet at the preinvitation stage of the CRC Screening Programme, but further research could be developed to elucidate if narratives, in another format and/or position, could positively impact uptake of the CRC screening programme overall and reduce inequalities.

Specifically, there is evidence that creating additional reminder letters is associated with increasing uptake [32, 33]. One potential advantage of using the narrative leaflet as part of an additional reminder would be that the leaflet could be used on its own rather than in combination with the standard informed choice leaflet. A RCT comparing the effectiveness of such a reminder with the narrative versus the standard leaflet would offer additional insights into the potential impact of our leaflet and circumvent the aforementioned limitations.

## 5. Conclusion

This pragmatic cluster randomised-trial found that providing a supplementary information leaflet, which presents a narrative account of the gFOBt-based, organised NHS CRC screening programme in England, neither increased overall uptake nor reduced inequalities, when delivered with the standard information material. This is despite the leaflet previously being found to significantly increase intentions to participate in CRC screening. The results do not support a change in policy at this time, but future research could investigate the impact of a stand-alone narrative leaflet at other points in the communication pathway.

## Supplementary Material

Supplementary Appendix 1 is an illustration of the A4 tri-fold narrative leaflet provided to the intervention group. The top and bottom sections show the outside and inside pages respectively.

## Figures and Tables

**Table 1:** Baseline characteristics.

	SI	SI + N
	*N* = 76,695	*N* = 73,722
	Median (range)	Median (range)

Age at invitation (in years)	65.0 (59.0–74.0)	65.0 (59.0–74.0)
IMD deprivation score	15.1 (0.5–87.8)	15.1 (0.5–87.8)

	% (*n*)	% (*n*)

Gender		
Female	51.0 (39,086)	51.5 (37,937)
Male	49.0 (37,609)	48.5 (35,785)
IMD quintile		
1st quintile (0–8.49, least deprived)	22.3 (17,073)	23.2 (17,027)
2nd quintile (8.50–13.79)	23.1 (17,675)	22.5 (16,517)
3rd quintile (13.80–21.35)	21.1 (16,161)	20.8 (15,287)
4th quintile (21.36–34.17)	17.5 (13,385)	17.6 (12,897)
5th quintile (34.18–87.80, most deprived)	15.9 (12,127)	16.0 (11,722)
*Missing*	*274*	*272*
Hub		
Midlands and North West	27.5 (21,118)	29.1 (21,421)
Southern	21.8 (16,723)	28.0 (20,667)
London	11.5 (8,795)	11.5 (8,509)
North East	16.8 (12,900)	17.7 (13,053)
Eastern	22.4 (17,159)	13.7 (10,072)
Screening round		
Prevalent first time invitees	16.3 (12,510)	20.7 (15,281)
Prevalent previous nonresponders	29.8 (22,892)	30.1 (22,209)
Incident episodes	53.8 (41,293)	49.1 (36,232)

SI: standard information; SI + N: standard information plus narrative leaflet; IMD: index of multiple deprivation.

**Table 2:** Proportion of individuals invited who were adequately screened^†^, according to gender, age, IMD quintile^‡^, hub, and screening round.

	SI	SI + N	Adjusted odds ratio	*p* value
	% (*n*)	% (*n*)	(95% CI)	
Total	58.5 (44,904)	56.7 (41,822)	1.00 (0.96–1.03)	0.80
Gender				
Female	60.9 (23,811)	59.3 (22,499)	1.01 (0.96–1.05)	0.77
Male	56.1 (21,093)	54.0 (19,323)	0.98 (0.94–1.03)	0.50
Age (median split)				
<65 years	55.2 (19,014)	53.3 (18,264)	1.01 (0.97–1.05)	0.67
≥65 years	61.2 (25,890)	59.7 (23,558)	0.98 (0.92–1.04)	0.45
IMD quintile				
1st quintile (0–8.49, least deprived)	66.8 (11,411)	64.6 (11,005)	0.98 (0.93–1.04)	0.57
2nd quintile (8.50–13.79)	62.7 (11,080)	62.1 (10,253)	1.00 (0.94–1.06)	0.91
3rd quintile (13.80–21.35)	59.4 (9,601)	58.3 (8,911)	1.05 (0.97–1.13)	0.24
4th quintile (21.36–34.17)	52.9 (7,083)	50.7 (6,535)	1.00 (0.94–1.06)	0.95
5th quintile (34.18–87.80, most deprived)	46.0 (5,580)	42.4 (4,966)	0.92 (0.86–0.98)	0.02
Hub				
Midlands and North West	57.6 (12,163)	53.4 (11,439)	0.95 (0.88–1.03)	0.22
Southern	60.2 (10,069)	61.5 (12,712)	1.00 (0.91–1.10)	0.98
London	49.2 (4,327)	45.4 (3,864)	1.00 (0.95–1.05)	0.95
North East	60.8 (7,837)	59.1 (7,716)	0.98 (0.90–1.06)	0.56
Eastern	61.2 (10,508)	60.5 (6,091)	1.07 (0.99–1.16)	0.08
Screening round				
Prevalent first time invitees	49.8 (6,231)	50.2 (7,678)	1.03 (0.99–1.08)	0.14
Prevalent previous nonresponders	14.3 (3,284)	14.0 (3,113)	0.97 (0.90–1.04)	0.35
Incident episodes	85.7 (35,389)	85.6 (31,031)	0.99 (0.94–1.04)	0.64

SI: standard information; SI + N: standard information plus narrative leaflet

^†^Returned a gFOBt kit (or kits) within 18 weeks of being sent the invitation, with a definitive “normal” (no further investigation required) or “abnormal” (requiring referral for further investigation, usually colonoscopy) screening test result available at the time of data extraction.

^‡^546 (274 SI and 272 SI + N) individuals missing an IMD score, 301 of these were adequately screened (149 SI and 152 SI + N).

**Table 3:** Proportion of individuals who were adequately screened^†^, according to IMD quintile^‡^ and median age at invitation.

	Age at invitation <65 years		Age at invitation ≥65 years	
	SI	SI + N	SI	SI + N
	*N* = 34,415	*N* = 34,260	*N* = 42,280	*N* = 39,462
	% (*n*)	% (*n*)	% (*n*)	% (*n*)

Adequately screened	55.2 (19,014)	53.3 (18,264)	61.2 (25,890)	59.7 (23,558)

1st quintile (least deprived)	64.4 (4,801)	61.4 (4,639)	68.7 (6,610)	67.2 (6,366)
2nd quintile	59.3 (4,577)	59.6 (4,424)	65.3 (6,503)	64.1 (5,829)
3rd quintile	55.6 (4,045)	55.1 (3,907)	62.5 (5,556)	61.0 (5,004)
4th quintile	49.4 (3,043)	47.1 (2,955)	55.9 (4,040)	54.1 (3,580)
5th quintile (most deprived)	43.5 (2,471)	39.3 (2,267)	48.3 (3,109)	45.4 (2,699)

SI: standard information; SI + N: standard information plus narrative leaflet.

^†^Returned a gFOBt kit (or kits) within 18 weeks of being sent the invitation, with a definitive “normal” (no further investigation required) or “abnormal” (requiring referral for further investigation, usually colonoscopy) screening test result available at the time of data extraction.

^‡^546 (274 SI and 272 SI + N) individuals missing an IMD score, 301 of these were adequately screened (149 SI and 152 SI + N).

**Table 4:** Proportion of individuals who were adequately screened^†^, according to IMD quintile^‡^ and gender.

	Males		Females	
	SI	SI + N	SI	SI + N
	*N* = 37,609	*N* = 35,785	*N* = 39,086	*N* = 37,937
	% (*n*)	% (*n*)	% (*n*)	% (*n*)

Adequately screened	56.1 (21,093)	54.0 (19,323)	60.9 (23,811)	59.3 (22,499)

1st quintile (least deprived)	63.9 (5,342)	61.2 (5,014)	69.7 (6,069)	67.8 (5,991)
2nd quintile	59.8 (5,186)	59.6 (4,757)	65.5 (5,894)	64.4 (5,496)
3rd quintile	56.7 (4,460)	55.6 (4,055)	61.9 (5,141)	60.7 (4,856)
4th quintile	50.8 (3,353)	48.1 (3,029)	55.0 (3,730)	53.1 (3,506)
5th quintile (most deprived)	44.7 (2,676)	40.7 (2,394)	47.3 (2,904)	44.1 (2,572)

SI: standard information; SI + N: standard information plus narrative leaflet.

^†^Returned a gFOBt kit (or kits) within 18 weeks of being sent the invitation, with a definitive “normal” (no further investigation required) or “abnormal” (requiring referral for further investigation, usually colonoscopy) screening test result available at the time of data extraction.

^‡^546 (274 SI and 272 SI + N) individuals missing an IMD score, 301 of these were adequately screened (149 SI and 152 SI + N).

**Table 5:** Proportion of individuals who were adequately screened^†^, according to IMD quintile^‡^ and screening round.

	Prevalent first time invitees		Prevalent previous nonresponders		Incident episodes	
	SI	SI + N	SI	SI + N	SI	SI + N
	*N* = 12,510	*N* = 15,281	*N* = 22,892	*N* = 22,209	*N* = 41293	*N* = 36232
	% (*n*)	% (*n*)	% (*n*)	% (*n*)	% (*n*)	% (*n*)

Adequately screened	49.8 (6,231)	50.2 (7,678)	14.3 (3,284)	14.0 (3,113)	85.7 (35,389)	85.6 (31,031)

1st quintile (least deprived)	58.0 (1,585)	58.9 (1,953)	17.8 (705)	16.3 (685)	87.9 (9,121)	88.0 (8,367)
2nd quintile	54.5 (1,494)	55.9 (1,827)	16.1 (775)	16.3 (705)	87.2 (8,811)	86.7 (7,721)
3rd quintile	50.5 (1,312)	51.4 (1,601)	14.2 (665)	15.7 (709)	85.7 (7,624)	86.1 (6,601)
4th quintile	44.7 (1,011)	44.4 (1,282)	13.1 (598)	12.5 (552)	83.7 (5,474)	84.1 (4,701)
5th quintile (most deprived)	37.9 (801)	37.3 (979)	11.1 (527)	9.6 (448)	81.0 (4,252)	79.8 (3,539)

SI: standard information; SI + N: standard information plus narrative leaflet.

^†^Returned a gFOBt kit (or kits) within 18 weeks of being sent the invitation, with a definitive “normal” (no further investigation required) or “abnormal” (requiring referral for further investigation, usually colonoscopy) screening test result available at the time of data extraction.

^‡^546 (274 SI and 272 SI + N) individuals missing an IMD score, 301 of these were adequately screened (149 SI and 152 SI + N).

**Table 6:** Proportion of individuals who were adequately screened^†^, according to IMD quintile^‡^ and hub.

	Midlands and North West		Southern		London		North East		Eastern	
	SI	SI + N	SI	SI + N	SI	SI + N	SI	SI + N	SI	SI + N
	*N* = 21,118	*N* = 21,421	*N* = 16,723	*N* = 20,667	*N* = 8,795	*N* = 8,509	*N* = 12,900	*N* = 13,053	*N* = 17,159	*N* = 10,072
	% (*n*)	% (*n*)	% (*n*)	% (*n*)	% (*n*)	% (*n*)	% (*n*)	% (*n*)	% (*n*)	% (*n*)

Adequately screened	57.6 (12,163)	53.4 (11,439)	60.2 (10,069)	61.5 (12,712)	49.2 (4,327)	45.4 (3,864)	60.8 (7,837)	59.1 (7,716)	61.2 (10,508)	60.5 (6,091)

1st quintile (least deprived)	66.9 (2,628)	62.2 (2,409)	65.1 (3,345)	65.4 (4,523)	63.9 (683)	56.1 (573)	69.1 (1,471)	68.8 (1,476)	68.3 (3,284)	65.8 (2,024)
2nd quintile	62.8 (2,859)	60.4 (2,674)	62.3 (2,666)	63.8 (3,287)	55.3 (839)	51.6 (778)	66.8 (1,841)	65.7 (1,822)	62.8 (2,875)	63.7 (1,692)
3rd quintile	60.3 (2,528)	56.3 (2,370)	58.9 (2,330)	61.2 (2,843)	50.8 (907)	49.2 (908)	63.7 (1,579)	62.9 (1,591)	60.1 (2,257)	58.2 (1,199)
4th quintile	53.3 (1,926)	49.1 (1,832)	53.0 (1,222)	52.7 (1,431)	44.6 (1,068)	41.7 (1,020)	58.0 (1,455)	56.6 (1,440)	55.1 (1,412)	55.8 (812)
5th quintile (most deprived)	45.9 (2,195)	41.5 (2,119)	47.4 (448)	49.8 (568)	40.7 (815)	34.5 (572)	49.1 (1,474)	45.2 (1,368)	46.3 (648)	43.0 (339)

SI: standard information; SI + N: standard information plus narrative leaflet.

^†^Returned a gFOBt kit (or kits) within 18 weeks of being sent the invitation, with a definitive “normal” (no further investigation required) or “abnormal” (requiring referral for further investigation, usually colonoscopy) screening test result available at the time of data extraction.

^‡^546 (274 SI and 272 SI + N) individuals missing an IMD score, 301 of these were adequately screened (149 SI and 152 SI + N).
