# Supplementary material for: Reducing the Social Gradient in Uptake of the NHS Colorectal Cancer Screening Programme Using a Narrative-Based Information Leaflet: A Cluster-Randomised Trial
Source: Gastroenterol Res Pract. 2016 Mar 16;2016:3670150. doi: 10.1155/2016/3670150 (PMC4812359; doi:10.1155/2016/3670150)
Supplement: Supplementary file 1 — Supplementary Appendix 1 is an illustration of the A4 tri-fold narrative leaflet provided to the intervention group. The top and bottom sections show the outside and inside pages respectively. [file 3670150.f1.pdf]

Screening stories

Judith's story

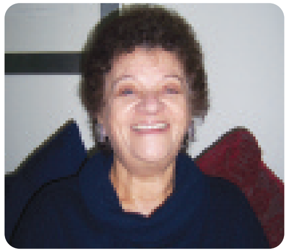

When the home-based test kit arrived Judith just put it to one side because she thought it was going to be too much work. However, when she did get around to doing it, she realised how quick and easy it was. Like most people, Judith got a 'normal' result, which she found to be very reassuring. For Judith, doing the test kit made her feel more in control of her health.

Chandulal's story

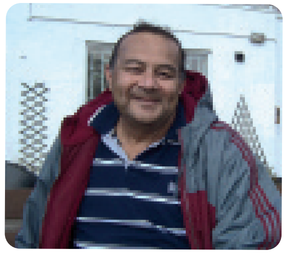

Chandulal did the test kit soon after it arrived through his letterbox. The results were 'abnormal' so he went to have a follow-up investigation at the bowel cancer screening centre. A few small growths were found in his bowel and removed for further tests. Much to his relief, they were found to be clear of cancer. Removing these growths was still very important because they could have turned into cancer over time. He now tells his friends that doing the test kit is simple and nothing to worry about.

Please keep this leaflet.
You may want to look over it again when the test kit arrives.

For more information please read "Bowel Cancer Screening: The Facts" (a copy was sent with this leaflet).

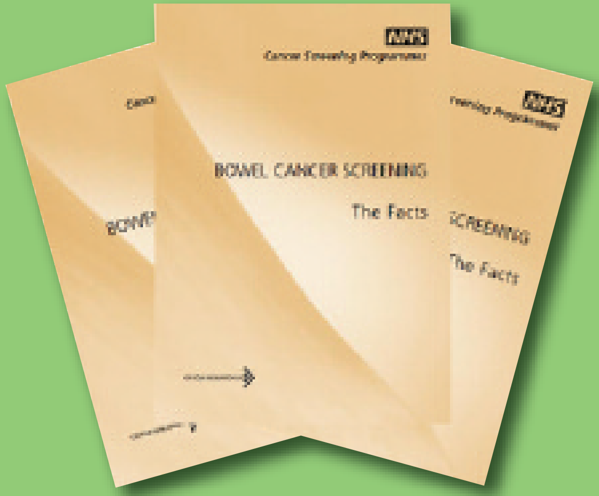

This leaflet was developed by University College London with funding from the National Institute for Health Research's programme grants for applied health research.

We would like to thank everyone who shared their experience of bowel cancer screening.

ASC NARR/03/13

Bowel Cancer Screening

People's Stories

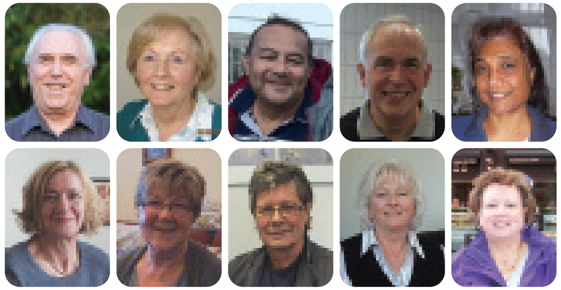

This leaflet tells you about other people's experiences of the English NHS Bowel Cancer Screening Programme

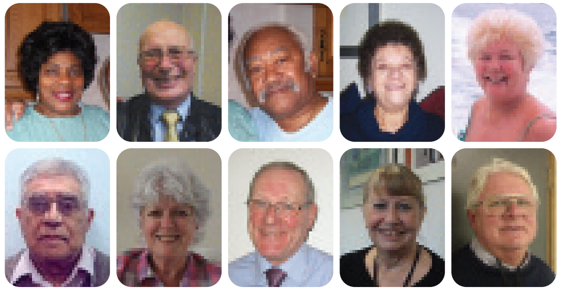

NHS
National Institute for Health Research

The screening programme aims to find bowel cancer early

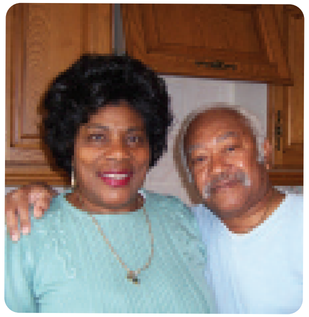

"It is just like having breast screening because it can pick up whatever is wrong before it develops into something bigger. If the doctors find something early it can be dealt with, and if they don't find anything then you're happy."

(Hyacinth)

The screening programme uses a home-based test kit which most people find easy to use

"My first thought about the test kit was that it was going to be messy, but it didn't actually turn out to be."

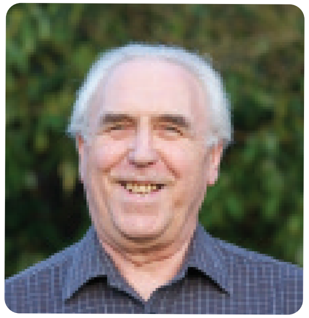

(Roger)

Most people (98 out of 100) will get a 'normal' result from the test kit

"When I got my reply to say everything was clear I was delighted. It was such a relief."

(Cynthia)

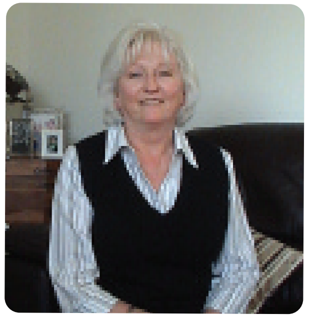

A small number of people (2 out of 100) get an 'abnormal' result and are offered a follow-up investigation

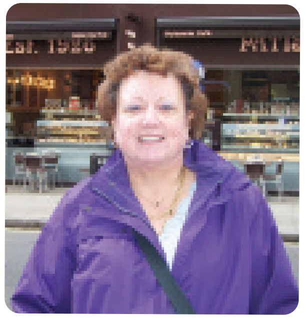

"I went and spoke to a very nice lady who explained that even though people get a call back they don't often get a cancer result. I felt a lot calmer after the appointment. I had the follow-up investigation the following Monday. They found two growths, which were removed. The results were fine and everything was ok."

(Monica)

Bowel cancer often has no early warning signs

"I was very lucky to have had the cancer picked up through screening. I had no symptoms at all so I would not have known anything was wrong. By the time I had got any symptoms, it would probably have been a lot more serious."

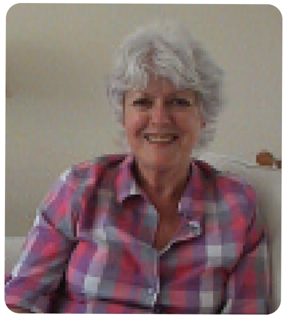

(Maureen)

Bowel cancer found through the screening programme is likely to be at an early stage and can be successfully treated

"The decision I made to complete the test kit was probably the best decision I have ever made in my life. Had I not taken that course of action, there is no doubt in my mind I would not be alive today."

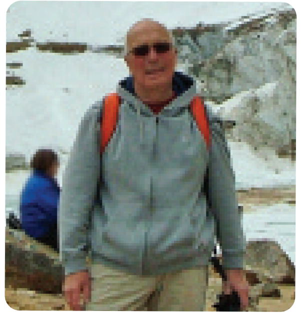

(Harold)
